# Rhabdomyolysis Induced by Coadministration of Fusidic Acid and Atorvastatin: A Case Report and Comprehensive Review of the Literature

**DOI:** 10.1155/2017/2187264

**Published:** 2017-10-08

**Authors:** Dimitrios Patoulias, Theodoros Michailidis, Thomas Papatolios, Rafael Papadopoulos, Petros Keryttopoulos

**Affiliations:** ^1^Department of Internal Medicine, General Hospital of Veria, Veria, Greece; ^2^Department of Nephrology, General Hospital of Veria, Veria, Greece

## Abstract

Statins are among the most widely prescribed medications worldwide. Acute rhabdomyolysis constitutes a potentially life-threatening side effect regardless of whether statins are administered alone or in combination. The potentially fatal combination of a statin and fusidic acid has been well described in the literature. Acute renal failure can be a direct consequence of this drug-drug interaction. We present a case of a 79-year-old woman who presented to our Emergency Department with a one-week history of limb weakness, myalgia, and inability to stand and walk. The patient had been given fusidic acid to treat Methicillin-Sensitive* Staphylococcus Aureus* (MSSA) positive dermatitis in the 3 weeks prior to admission, while she continued to take her complete therapeutic regimen, which included atorvastatin. Thus, she developed rhabdomyolysis due to the interaction between fusidic acid and atorvastatin. Herein, we report a life-threatening complication of coadministration of fusidic acid and a statin, which is preventable and predictable. The exact mechanism of the interaction is not fully understood, but coadministration of these two medications must be avoided in clinical practice.

## 1. Purpose

The purpose of the present case study is to highlight rhabdomyolysis as a potentially life-threatening consequence after coadministration of fusidic acid and a statin. Although the exact mechanism of interaction is not fully understood, the coadministration must be avoided in clinical practice. We present a case of rhabdomyolysis after prescription of fusidic acid in a patient previously treated with atorvastatin who developed rhabdomyolysis early after the coadministration.

## 2. Case Report

A 79-year-old female patient was admitted to the Emergency Department on 21 September 2016 complaining of one-week history of diffuse limb myalgia and inability to stand or walk. Her medical history was notable for arterial hypertension, type 2 diabetes mellitus, chronic kidney disease, and dyslipidemia over the last 25, 15, 6, and 10 years, respectively. She underwent a right nephrectomy in 2013 for renal oncocytoma.

She had a three-month history of MSSA positive dermatitis of the right knee, which was initially treated with topical antibiotics. Due to inadequate response to treatment, she was prescribed a 3-week course of fusidic acid 250 mg four times daily and clindamycin 300 mg three times daily. At the same time, the patient remained on her therapeutic regimen, which included atorvastatin 20 mg once daily.

During the initial assessment of the patient, her vital signs were within normal range. Her physical examination was unremarkable, except for the significant decrease in muscle strength of the proximal leg muscles. Abnormal laboratory values were as follows: SGOT, 635 U/L; SGPT, 317 U/L; LDH, 1335 U/L; CPK, 17263 U/L; CK-MB, 534 U/L; TBIL, 1.54 mg/dl; DBIL, 0.91 mg/dl; IBIL, 0.63 mg/dl; urea, 121 mg/dl; and Crea., 2.6 mg/dl. Air blood gases test revealed the following: Ph, 7.45; PO_2_, 79.7 mmHg; PCO_2_, 37.9 mmHg; and HCO_3_, 26.0 mmol/l. Electrolytes were all within the normal range. Myoglobinuria was also present, while there was no hematuria. Abdominal ultrasonography did not reveal any pathology. Computerized tomography of the brain was also unremarkable.

She had no history of trauma, significant exertion, or immobilization. Screening for autoimmune diseases, including ANA, anti-dsDNA, c-ANCA, p-ANCA, and anti-Jo-1, was negative. The patient did not give a written consent for the performance of a muscle biopsy in order to confirm the diagnosis histologically. She was finally diagnosed with acute rhabdomyolysis due to coadministration of fusidic acid and atorvastatin.

Intravenous hydration and diuresis were the only therapeutic intervention, while sodium bicarbonate was administered only on the first day of hospitalization. Antibiotic treatment and statin therapy were discontinued.

The range of the main laboratory values of the patient during hospitalization is shown in Table 1.

The patient gradually regained muscle strength and was able to stand and walk before being discharged home. Ten days later, her CPK, SGOT, and SGPT values had normalized, while her serum creatinine level was at baseline. During her follow-up on a 3-month basis, she remains asymptomatic without issues.

## 3. Discussion

Few case reports highlighting the potentially life-threatening drug-drug interaction between statins and fusidic acid have been published (Table 2).

Magee et al. [1] published a series of 4 cases of acute rhabdomyolysis after coadministration of fusidic acid and atorvastatin. All patients were on long-term therapy with atorvastatin before administration of fusidic acid. Fusidic acid was prescribed due to osteomyelitis in three patients and septic arthritis in one patient. Two out of three patients suffered previously from end-stage renal disease and diabetes mellitus type 2. They died after admission to ICU due to multiple organ dysfunction syndrome (MODS). The third patient suffered from diabetes mellitus type 1. He developed acute kidney injury in the term of rhabdomyolysis. The 4th patient developed acute kidney injury due to rhabdomyolysis, but aggressive measures led to normalization of his renal function. It is notable that he was the younger patient in this case series, with no significant medical history. The authors emphasize on the need of avoidance of coadministration of the two agents.

In the largest available case series including 8 patients who developed rhabdomyolysis due to interaction of statin and fusidic acid, Kearney et al. [2] conclude the following: (a) the risk of rhabdomyolysis is 50-fold higher in patients receiving both statin and fusidic acid compared with patients receiving only a statin, (b) admission to ICU in those cases may be required, especially in patients with subject diseases, (c) MODS is the typical cause of death, and (d) long-term follow-up of those patients is usually required, with emphasis on their renal function. The authors suggest that, in cases of short-term therapy with fusidic acid, statin should be temporarily withdrawn, while when long-term therapy is required statin should be replaced by another lipid-lowering agent.

Another case series of rhabdomyolysis induced by interaction of fusidic acid and statin was presented by Collidge et al. [3]. The authors report 4 cases of patients suffering from diabetes mellitus type 2 who were prescribed the discussed combination. Three of them received simvastatin, and one received atorvastatin. Rhabdomyolysis was diagnosed at least two weeks after initiation of fusidic acid (18–32 days). The authors emphasize upon the potential mimicry between rhabdomyolysis and Guillain-Barré Syndrome (GBS). All 4 patients were initially diagnosed with GBS due to clinical presentation, nerve conduction studies, and needle electromyogram. The combination was discontinued 0–2 days after notice of elevated serum CK levels. Long rehabilitation and gradual restoration of patients' mobility were required.

The exact mechanism of interaction between the two agents remains unclear. According to previous reports, fusidic acid serves as a CYP450 3A4 enzyme inhibitor [4–7]. However, the latter has been questioned [1]. Based on the fact that the main metabolite of fusidic acid is a glucuronide conjugate, interference with the glucuronidation system potentially leads to increased serum levels of statins and thus significant risk of myotoxicity. According to Eng et al. [8], fusidic acid inhibits significantly organic anion transporting polypeptides OATP1B1 and OATP1B3, modifying hepatic uptake of statins, leading to increased serum concentration and, thus, increased risk of rhabdomyolysis. In the same study, fusidic acid was recognized as a weak, reversible, and time-dependent inhibitor of CYP450 3A4, while it did not affect breast cancer resistance protein (BCRP). In the study performed by Gupta et al. [9], the researchers confirmed that fusidic acid inhibits BCRP and OATP1B1 mediated liver and intestinal uptake of statins, while it also inhibits hepatic metabolism of statins. According to the authors, single dosing of fusidic acid leads to unbound plasma or hepatic inlet levels high enough to inhibit OATP1B1 and P450s, while multiple dosing of fusidic acid, as in all cases described above, inhibits BCRP as well. Despite the interesting findings of Eng et al. and the perspective of Gupta et al., it is clear that further studies are required in order to elucidate the mechanisms of this drug-drug interaction.

## 4. Conclusions

We conclude the following:

1. The mechanism of interaction between statins and fusidic acid is under discussion. Further investigation is required.
2. Acute kidney injury is the main cause of morbidity and mortality in patients with rhabdomyolysis after coadministration of fusidic acid and a statin. Certain factors (medical history, other medications, etc.) may worsen the clinical outcome.
3. Coprescription of the two agents must be avoided in clinical practice. This drug-drug interaction is preventable and predictable.

## Figures and Tables

**Table 1:** 

	1st day	2nd day	4th day	5th day	6th day	7th day	8th day	11th day	14th day	17th day	21st day
CPK (U/L)	17263	11632	12094	9835	13287	9104	4491	2231	987	556	227
Urea (mg/dl)	121	99	96	90	85	86	79	74	72	71	70
Crea. (mg/dl)	2.6	1.86	1.4	1.69	1.58	1.58	1.58	1.53	1.51	1.52	1.51
SGOT (U/L)	635	541	552	524	574	440	251	117	94	65	44
SGPT (U/L)	317	284	318	356	401	391	322	163	123	87	57

**Table 2:** Summary of the published case reports highlighting the discussed drug-drug interaction.

Authors	Statin	Fusidic acid in therapeutic dosage	Indication for fusidic acid administration	Outcome
Bachoumas et al. [10]	Pravastatin	✓	MSSA positive abscess of the tibial plate	Death

Wenisch et al. [11]	Atorvastatin	✓	*S. aureus* and *E. coli*-infected gangrenous foot lesion	Discharged home with restored mobility

Gabignon et al. [12]	Atorvastatin	✓	MSSA positive hip prosthetic joint infection	Discharged home with restored mobility

Nandy and Gaïni [13]	Atorvastatin	✓	Infected aortic aneurysm, with involvement of the endovascular stent and para-aortic abscesses	Discharged home with improved mobility

Cowan et al. [4]	Atorvastatin/rosuvastatin	✓	MSSA positive hip prosthesis	Death

Burtenshaw et al. [14]	Simvastatin	✓	Isolation of MRSA from aneurysmal sac after elective AAA repair	Death

Teckchandani et al. [5]	Atorvastatin	✓	Infected hip prosthesis	Discharged home with restored mobility

O'Mahony et al. [15]	Atorvastatin	✓	NP	Discharged home with restored mobility

Saeed and Azam [6]	Atorvastatin	✓	Postarthroscopy MRSA right knee infection	Discharged home with improved mobility

NP: not provided.
